# Inhibition of TIGIT on NK cells improves their cytotoxicity and HIV reservoir eradication potential

**DOI:** 10.1128/mbio.03226-24

**Published:** 2025-02-07

**Authors:** Yue Wang, Yidi Li, Jiaqi Chen, Chenxi Guo, Xiaowen Yu, Zining Zhang, Yajing Fu, Xiaoxu Han, Qinghai Hu, Haibo Ding, Hong Shang, Yongjun Jiang

**Affiliations:** 1 State Key Laboratory for Diagnosis and Treatment of Infectious Diseases, NHC Key Laboratory of AIDS Prevention and Treatment, National Clinical Research Center for Laboratory Medicine, The First Hospital of China Medical University, China Medical University, Shenyang, China; 2 Key Laboratory of AIDS Immunology, Chinese Academy of Medical Sciences, Shenyang, China; 3 Key Laboratory of AIDS Immunology of Liaoning Province, Shenyang, China; 4 Collaborative Innovation Center for Diagnosis and Treatment of Infectious Diseases, Hangzhou, China; University of Pittsburgh School of Medicine, Pittsburgh, Pennsylvania, USA; University of Pittsburgh, Pittsburgh, Pennsylvania, USA

**Keywords:** HIV reservoir, NK cells, TIGIT, IFN-γ

## Abstract

The latent human immunodeficiency virus (HIV) reservoir presents the biggest obstacle to curing HIV chronic infection. Consequently, finding novel strategies to control the HIV reservoir is critical. Natural killer (NK) cells are essential for antiviral immunity. However, the influence of NK cell subsets and their associated inhibitory or activating receptors on their cytotoxicity toward the HIV reservoir has not been fully studied. We investigated the relationship between the percentage of NK cells or NK cell subsets and the HIV reservoir. Our results indicated that the percentage of CD56^−^CD16^+^ NK cells was positively associated with HIV reservoir size (i.e., HIV DNA, HIV msRNA, or HIV usRNA). Additionally, we observed that the percentage of IFN-γ^+^ NK cells was inversely related to the HIV reservoir. Furthermore, the expression of TIGIT on NK cells, particularly CD56^-^CD16^+^ and CD56^dim^ NK cell subsets, positively correlated with the HIV reservoir. Notably, individuals with higher percentage of TIGIT^+^ NK and lower percentage of CD226^+^ NK cells exhibited larger HIV reservoir. Mechanistically, we discovered that TIGIT could inhibit the PI3K-Akt-mTOR-mTORC1 (s6k) signaling pathway to decrease the production of IFN-γ in NK cells. Importantly, inhibiting TIGIT in NK cells enhanced their ability to eliminate reactivated latently infected CD4^+^ T cells. Our experiments underscored the crucial role of NK cells in controlling the HIV reservoir and suggested that TIGIT serves as a promising target for enhancing the NK cell-mediated clearance of the HIV reservoir.

**IMPORTANCE:**

As a major barrier to human immunodeficiency virus (HIV) cure, HIV reservoir persist in viremia-suppressed infected individuals. NK cells are important antiviral cells, and their impact on reservoir has rarely been reported. We analyzed the relationship between the size of reservoir and NK cell subsets, inhibitory receptor TIGIT expression. Our analysis found that the percentage of CD56^−^CD16^+^ NK cells was positively associated with HIV reservoir size. Furthermore, TIGIT expression on NK cells and CD56^−^CD16^+^ NK cells or CD56^dim^ NK cells has a positive correlation with the HIV reservoir. TIGIT can inhibit the PI3K-Akt-mTOR-mTORC1 (s6k) signaling pathway to decrease the production of IFN-γ on NK cells. Blocking TIGIT in NK cells can enhance their ability to eliminate reactivated latently infected CD4^+^ T cells. Our study indicated that NK cells are critical to the control of the reservoir size, and TIGIT may be a target for enhancing the NK cell-mediated elimination of the reservoir.

## INTRODUCTION

Chronic HIV infection caused by the human immunodeficiency virus (HIV) has long been a problematic issue. With the advent of antiretroviral therapy (ART), HIV infection has gradually transitioned from a fatal disease to a chronic and manageable one. The initiation of ART has been shown to reduce plasma viral loads to below the clinical detection limit, as well as decrease the number of cells in the blood and tissues that harbor HIV DNA (1). However, ART fails to eliminate HIV from the body because HIV integrates its transcriptionally silent DNA into the host genes, where it persists in latently infected CD4^+^ T cells (the latent HIV reservoir). Latent host cells that are reactivated by antigens or ART interruption can produce infectious virus (2). Furthermore, low-level HIV transcription and viral protein production can be detected in a fraction of cells during ART. These cells are considered the active HIV reservoir (3, 4) and can generally be identified by measuring levels of HIV multiply spliced RNA (msRNA) and HIV unspliced RNA (usRNA) levels. These transcriptionally and translationally active reservoirs may be responsible for the sustained immune activation and cellular exhaustion observed in ART-treated patients (5, 6).

The latent HIV reservoir represents the most significant obstacle in the pursuit of an HIV cure. Virus-specific CD8^+^ T cells are capable of controlling HIV viremia (7), yet cytotoxic T lymphocytes (CTLs) may play a limited role in eradicating the HIV reservoir. Infected individuals who achieve viremia suppression after ART have weaker CTL responses than elite controllers (8). Furthermore, the intensity of HIV-specific CTL response levels positively correlates with HIV DNA in infected individuals undergoing long-term ART, indicating that CTLs can respond to infected cells but may be unable to eliminate them (9). Therefore, there is a pressing need to explore other factors that influence the HIV reservoir to devise innovative strategies aimed at curing HIV.

Natural killer (NK) cells are vital for the innate immune response (10), capable of eliminating tumors and virally infected cells without the need for specific antigen stimulation (11, 12). NK cells can be categorized into three subsets on account of their differential expression of CD56 and CD16: CD56^dim^ and CD56^bright^ (13) and CD56^−^CD16^+^ NK cell subsets (14). The role of NK cells in controlling HIV infection and their therapeutic potential has been recognized for a long time (15). Previous studies have found that viremic controllers exhibited a higher percentage of NK cells than non-controllers (16) and individuals with a lower viral set-point had a higher percentage of NK cells during primary infection (17). However, the CD56^−^CD16^+^ NK cell subset has been proven to play a negative regulatory role (18). These studies are limited to exploring the control effect of NK cells on viremia. A previous study reported that during treatment with a latency reversal agent, the percentage of CD3^−^CD56^+^ and CD56^dim^CD16^+^ NK cells was inversely correlated with HIV-1 DNA (19). In addition, one study showed that the percentage of NK cells and CD16^+^ NK cells in particular was negatively correlated with total HIV DNA (20). However, the relationship between the percentage of NK cell subsets and the HIV active reservoir (HIV msRNA and HIV usRNA) remains to be further investigated. One study found a negative correlation between the percentage of IFN-γ^+^ NK cells and HIV DNA (21). Nevertheless, the relationship between the percentage of IFN-γ^+^ NK cells and the active reservoir (as indicated by HIV msRNA and HIV usRNA copy numbers) has yet to be reported. Additionally, several studies found that the expression of partially activated receptors that enhance NK cell function correlates with HIV reservoir size. For instance, the percentage of NKG2D^+^ and NKp30^+^ NK cells were inversely related to the size of the reservoir in hepatitis C virus (HCV)/HIV co-infected individuals on ART after IFN-α treatment (22). In contrast to activating receptors, inhibitory receptors exerted a negative effect on NK cell function. The T cell immunoreceptor with Ig and ITIM domains (TIGIT) is an important inhibitory receptor on NK cells. One study found that TIGIT was highly expressed in NK cells during HIV infection and inhibits NK cell function (23). Furthermore, TIGIT expression has been found to suppress the cytotoxicity of NK cells to PVR^+^P24^+^CD4^+^ T cells in HIV-infected individuals (24). However, the relationship between TIGIT expression on NK cells and the size of the HIV reservoir as well as the specific mechanism by which TIGIT affects NK cell function, remains unreported and requires further investigation.

In this study, we observed the relationship between the percentage of NK cells or individual NK subsets and HIV DNA concentration (latent HIV reservoir) or HIV msRNA and usRNA levels (active HIV reservoir). Specifically, we explored the association of the percentage of IFN-γ^+^ NK cells (and individual NK cell subsets) with latent and active HIV reservoir. Furthermore, we investigated the relationship between latent or active HIV reservoir and the TIGIT expression on NK cells. Additionally, we delved into the mechanisms underlying how TIGIT affects NK cell function. We also explored the cytotoxicity of NK cells against reactivating latent HIV-infected cells and the influence of blocking TIGIT receptors on NK cells. Ultimately, these works primarily focus on exploring the possibility of utilizing NK cells as effector cells for the elimination of the HIV reservoir.

## MATERIALS AND METHODS

### Study participants

In this study, 57 HIV-infected individuals from the First Hospital of China Medical University (CMU) were selected for investigation into HIV reservoir. All participants had been receiving ART for over 2 years and had achieved post-treatment viral loads of less than 40 copies/mL. The cohort was composed of 56 males and one female, with a median age of 35 years. These patients had a median ART duration of 48 months, and their median CD4^+^ T cell count was 597 cells/µL, as detailed in Table 1. Furthermore, an additional 14 HIV-infected individuals were enrolled to investigate the relationship between TIGIT and CD57 on NK cells. The Ethics Committee of the First Hospital of CMU approved all protocols.

**TABLE 1:** Characteristics of clinical study participants^*a*^

Characteristics	HIV (*n* = 57）
Age (years, median with range）	35 (21, 60）
Sex (female:male）	1:56
Time on ART (months, median with range）	48 (24,127）
CD4^+^ T cell counts (cells/mm³, median with range）	597 (220, 1273)

^*a*^ All participants had been receiving ART for over 2 years and have achieved post-treatment viral loads of less than 40 copies/mL.

### Detection of TIGIT on the surface of NK cells

Peripheral blood mononuclear cells (PBMCs) were isolated by Hypaque-Ficoll (GI, USA) from whole blood. The expression of TIGIT on NK cells was detected by a panel of fluorescent dye, including anti-CD3-PerCP (BioLegend, USA), anti-CD14-PerCP (BioLegend, USA), anti-CD19-PerCP (BioLegend, USA), 7AAD (BD Pharmingen, USA), anti-CD16-APCCy7 (BioLegend, USA), anti-CD56-PECy7 (BioLegend, USA), and anti-TIGIT-APC (BioLegend, USA). The NK cells were stratified into three subsets based on their expression levels of CD56 and CD16. These markers’ expression on NK cells was analyzed with flow cytometry (FACS LSR-II, BD Biosciences, USA).

### Detection of IFN-γ production by NK cells

PBMCs were incubated in 96-well round-bottom plates (Corning, USA) and stimulated with a combination of cytokines consisting of interleukin-12 (IL-12), IL-15, and IL-18 at 10, 50, and 100 ng/mL, respectively (IL-12/15/18) (R&D Systems, USA). The cells were cultured in RPMI media (HyClone, USA) containing 10% fetal bovine serum (Thermo Fisher, USA) and 1% penicillin (TBDscience, China) for 24 h. Golgi-stop (BD Biosciences, USA) was added 6 h before the cells were collected. After collection, the cells were washed then stained with Live/Dead dye (Thermo Fisher, USA), and incubated at 4°C for 30 min without light. Next, the cells were washed with phosphate-buffered saline (PBS, HyClone, USA) and stained with a panel of fluorescent dye, including anti-CD3-PerCP (BioLegend, USA), anti-CD14-PerCP (BioLegend, USA), anti-CD19-PerCP (BioLegend, USA), 7AAD (BD Pharmingen, USA), anti-CD16-APCCy7 (BioLegend, USA), anti-CD56-PECy7 (BioLegend, USA), and incubated at 4°C for 20 min without light. For intracellular staining, the cells were permeabilized using a permeabilization reagent (BD Biosciences, USA) and incubated at 4°C for 30 min without light. After washing with Perm buffer (BD Biosciences, USA), the cells were stained with anti-IFN-γ-APC (BioLegend, USA), and incubated at 4°C for 20 min without light. Finally, the cells were washed with Perm buffer and analyzed by flow cytometry (FACS LSR-II, BD Biosciences, USA).

### Nucleic acid extraction and RNA reverse transcription

For the isolation of CD4^+^ T cells from PBMCs, the EasySep Human CD4^+^ T Cell Isolation Kit (Stem Cell, USA) was employed. The DNA of CD4^+^ T cells was extracted using the QIAamp DNA Blood Mini Kit (QIAGEN, Germany). For RNA extraction, the RNeasy Plus Mini Kit (Qiagen, Germany) was used. The concentration and purity of each sample were detected by Nanodrop Lite Spectrophotometer (Thermo Fisher, USA). After that, the RNA was reverse-transcribed into cDNA using the iScript Advanced cDNA Synthesis Kit (Bio-Rad, USA). The reaction setup is detailed in Table S1. The reaction conditions consisted of an incubation at 42°C for 30 min, followed by a step at 85°C for 5 min, with the final step being a pause at 4°C.

### HIV reservoir detection with digital droplet PCR

The QX200 digital droplet PCR system (Bio-Rad, USA) was used to detect HIV total DNA, HIV usRNA, and HIV msRNA. HIV DNA serves as a biomarker for the total viral reservoir, which encompasses both cells capable of transcribing viral RNA and those incapable of doing so. Conversely, HIV msRNA or usRNA is a part of the total reservoir, which consists of cells that actively transcribe viral RNA (active HIV reservoir) (25). The DNA or cDNA template was introduced into the PCR reaction system (listed in Table S2), along with the primers and probes (listed in Table S3). The reaction mixture was then split into eight sample wells of the DG8 cartridge (Bio-Rad, USA). Subsequently, 70 µL of droplet-generating oil (Bio-Rad, USA) was added to the oil wells. The cartridge was inserted into a QX200 droplet generator (Bio-Rad, USA), and the droplets were transferred to a 96-well plate (Bio-Rad, USA) for PCR amplification. The PCR amplification was conducted under the following conditions: initial step at 95°C for 10 min, followed by 40 amplification cycles (94°C for 30 s and 60°C for 1 min) and a final extension step at 98°C for 10 min, with the reaction concluding at 16°C. The 96-well plate was finally transferred into the QX200 droplet reader and the size of the HIV reservoir was analyzed with the QX200 Droplet Digital PCR System (Bio-Rad, USA). The corresponding computational formula for this analysis is detailed in the article (26).

### Signaling pathway phosphorylation level detection

NK cells were negatively selected with EasySep Human NK Cell isolation kit (Stem Cell, USA) and then resuspended with 200 µL of sugar-free medium (Thermo, USA), and incubated for 1 h with CD155- Fc (5 µg/mL) (R&D Systems, USA) in the treatment tubes. BD Phosflow Fix Buffer I (BD Pharmingen, USA) was heated to 37°C and BD Phosflow Perm Buffer III (BD Pharmingen, USA) was cooled to −20°C for later use. After 1 h, the cells were stimulated with a combination of cytokines consisting of IL-12, IL-15, and IL-18 (10, 20, and 100 ng/mL IL-18) (R&D Systems, USA) for 45 min. After stimulation, immediately mix 200 µL of pre-warmed BD Phosflow Fix Buffer I (BD Pharmingen, USA) was added to the cells in the tube and incubated for 10 min in a 37°C water bath. After incubation, the cells were centrifuged to discard the supernatant and washed once with BD Pharmingen Stain Buffer (BD Pharmingen, USA). The cells were then vortexed to loosen them and 100 µL of pre-cold BD Phosflow Perm Buffer III (BD Pharmingen, USA) was added while vortexing to permeabilize the cells, and then the cells were incubated on ice for 30 min. The cells were washed two times with 3 mL BD Pharmingen Stain Buffer (BD Pharmingen, USA). Subsequently, anti-mTOR-PE (BD, USA) and anti-s6k-APC (Invitrogen, USA) dyes were added to the cells, which were then incubated at 4°C for 30 min without light. The cells were washed and analyzed with flow cytometry.

### Signaling pathway blocking experiment

PBMCs were extracted from HIV-infected patients and placed in 96-well plates. The treated wells were then incubated with Torin 1 (200 nmol/L) (MedChemExpress, USA) and CMK (50 µmol/L) (MedChemExpress, USA) for 1 h. After this incubation period, the cells were stimulated with the combination of cytokines consisting of IL-12, IL-15, and IL-18 (10, 20, and 100 ng/mL) (R&D Systems, USA). Unstimulated wells served as negative controls. The following steps for cell culture, surface staining, membrane rupture, and intracellular staining were followed as described in “Detection of IFN-γ production by NK cells.”

### Detection of the level of intracellular p24 in latent HIV-infected CD4^+^ T cells suppression assay

CD4^+^ T and NK cells were negatively selected with EasySep Human CD4^+^ T/ NK Cell isolation kits (STEM CELL, USA), respectively, and the cells were counted. The CD4^+^ T cells were stimulated by Dynabeads Human T-Activator CD3/CD28 (Thermo Fisher, USA) for 72 h. Prior to stimulation, the NK cells were pre-incubated with a functional grade purified anti-human TIGIT antibody (5 µg/mL; eBioscience, USA) for 1 h, followed by stimulation with a combination of cytokines for 72 h. The NK cells were then washed and co-cultured with the activated CD4^+^ T cells at a ratio of 1:2 for 5 h. After the co-culture, the cells were collected, treated with Live/Dead dye (Thermo Fisher, USA) for 30 min at 4°C without light. The cells were washed and stained with anti-CD3-PerCP (BioLegend, USA) and anti-CD4-APCCy7 (BioLegend, USA) for 20 min at 4°C without light. The cells were incubated with perm reagent (BD Biosciences, USA) for 30 min at 4°C without light. The cells were washed with Perm buffer (BD Biosciences, USA) and stained with anti-P24-APC (MEDIMABS, Canada) for 20 min at 4°C without light. Finally, the cells were washed and analyzed with flow cytometry.

### Detection of the level of HIV RNA in latent HIV-infected CD4^+^ T cells suppression assay

CD4^+^ T and NK cells were negatively selected with EasySep Human CD4^+^ T/NK Cell isolation kits (Stem Cell, USA), respectively, and the cells were counted. The CD4^+^ T cells were stimulated by Dynabeads Human T-Activator CD3/CD28 (Thermo Fisher, USA) for 72 h. Prior to stimulation, the NK cells were pre-incubated with a functional grade purified anti-human TIGIT antibody (5 µg/mL; eBioscience, USA) for 1 h, followed by stimulation with a combination of cytokines for 72 h. The NK cells were then washed and co-cultured with the activated CD4^+^ T cells at a ratio of 1:2 for 72 h. After the co-culture, the cells were collected, and then the QX200 digital droplet PCR system (Bio-Rad, USA) was used to detect HIV usRNA and HIV msRNA was followed as described in “HIV reservoir detection with digital droplet PCR.”

### Statistical analysis

GraphPad Prism 8 and SPSS20.0 software were used for statistical analysis of experimental results. To assess the normality of the data distributions, the Shapiro-Wilk test was applied. For comparisons between two independent groups with non-normal distributions, the nonparametric Mann–Whitney *U* test was utilized. For paired comparisons of non-normally distributed data, the Wilcoxon paired rank-sum test was employed. Furthermore, the Spearman rank correlation test was performed to analyze the relationship between two non-normally distributed data sets. False discovery rate (FDR) was used to correct multiple testing in correlation analyses. *P* < 0.05 was identified as statistically significant.

## RESULTS

### HIV-infected individuals with a higher percentage of CD56^dim^ NK cells have a smaller reservoir

**Fig 1:**
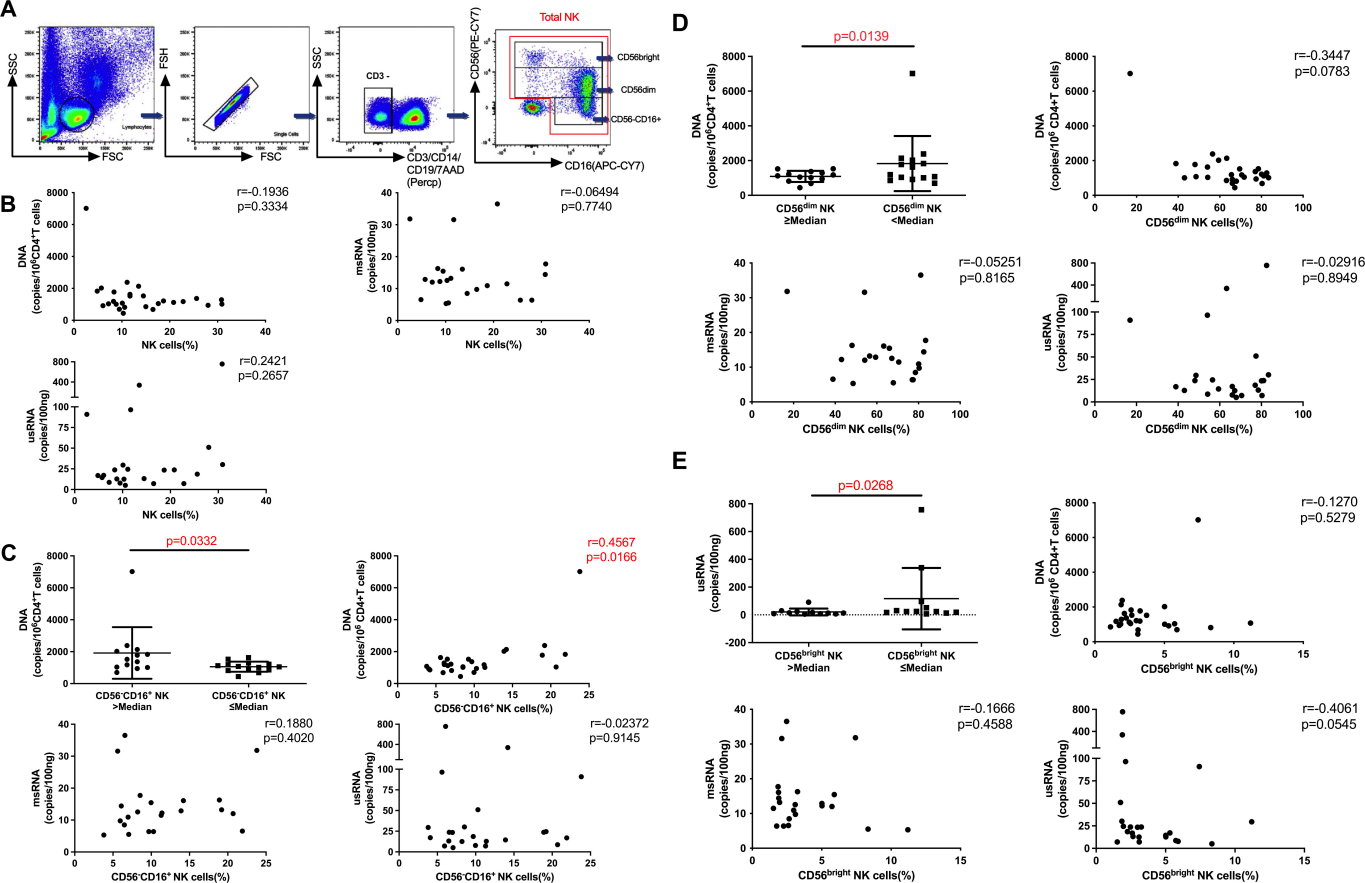
The relationship between the percentage of NK cells and NK subsets of HIV-infected individuals and the reservoir. (A) Gating strategy for flow cytometry of three NK subsets. (B) Correlation analysis between the percentage of NK cells and HIV DNA (*n* = 27), msRNA (*n* = 22), and usRNA (*n* = 23). (C) Comparison of HIV DNA between HIV-infected individuals with high and low percentages of CD56^−^CD16^+^ NK cells (*n* = 27), and the relationship analysis between the percentage of CD56^−^CD16^+^ NK cells and HIV DNA (*n* = 27), msRNA (*n* = 22), and usRNA (*n* = 23). (D) Comparison of HIV DNA between HIV-infected individuals with high and low percentages of CD56^dim^ NK cells (*n* = 27), and the relationship analysis between the percentage of CD56^dim^ NK cells and HIV DNA (*n* = 27), msRNA (*n* = 22), and usRNA (*n* = 23). (E) Comparison of HIV usRNA between HIV-infected individuals with high and low percentages of CD56^bright^ NK cells (*n* = 23), and the correlation analysis between the CD56^bright^ NK cell percentage and HIV DNA (*n* = 27), msRNA (*n* = 22), and usRNA (*n* = 23). Spearman correlation was used for correlation analysis, Mann–Whitney *U* test was used for comparison between the two groups.

We delved into the relationship between the percentage of total NK cells or each of these three NK subsets and the size of the HIV reservoir. The gating strategy employed to distinguish between the NK cell subsets is illustrated in Fig. 1A. The results noted that the percentage of total NK cells did not significantly correlate with HIV DNA, HIV msRNA, or HIV usRNA concentrations (Fig. 1B). A higher HIV DNA copy number was observed in a group with a high percentage of CD56^−^CD16^+^ NK cells (*P* = 0.0332), and the percentage of CD56^−^CD16^+^ NK cells was significantly positively associated with HIV DNA copy numbers (*r* = 0.4567, *P* = 0.0166). However, there was no significant relationship between the percentage of CD56^−^CD16^+^ NK cells and HIV msRNA or usRNA concentrations (Fig. 1C).

Lower HIV DNA copy numbers were detected in a group with a high percentage of CD56^dim^ NK cells (*P* = 0.0139); there was an inverse relationship trend between the percentage of CD56^dim^ and HIV DNA copy numbers (*r* = −0.3447, *P* = 0.0783). Similarly, no correlation was observed between the percentage of CD56^dim^ NK cells and the HIV msRNA or usRNA concentrations (Fig. 1D). Furthermore, a group with a high percentage of CD56^bright^ NK cells exhibited lower HIV usRNA concentrations (*P* = 0.0268); there was an inverse relationship trend between the percentage of CD56^bright^ and HIV usRNA concentrations (*r* = −0.4061, *P* = 0.0545). Nevertheless, the percentage of CD56^bright^ NK cells did not significantly correlate with HIV DNA or msRNA concentrations (Fig. 1E). The correction was done for multiple testing in correlation analyses with FDR analysis, the adjusted *P* values for the correlation between the percentage of CD56^−^CD16^+^ NK cells with HIV DNA copy numbers were not significant.

### The percentage of IFN-γ^+^ NK cells of HIV-infected individuals have a negative correlation with HIV reservoir

**Fig 2:**
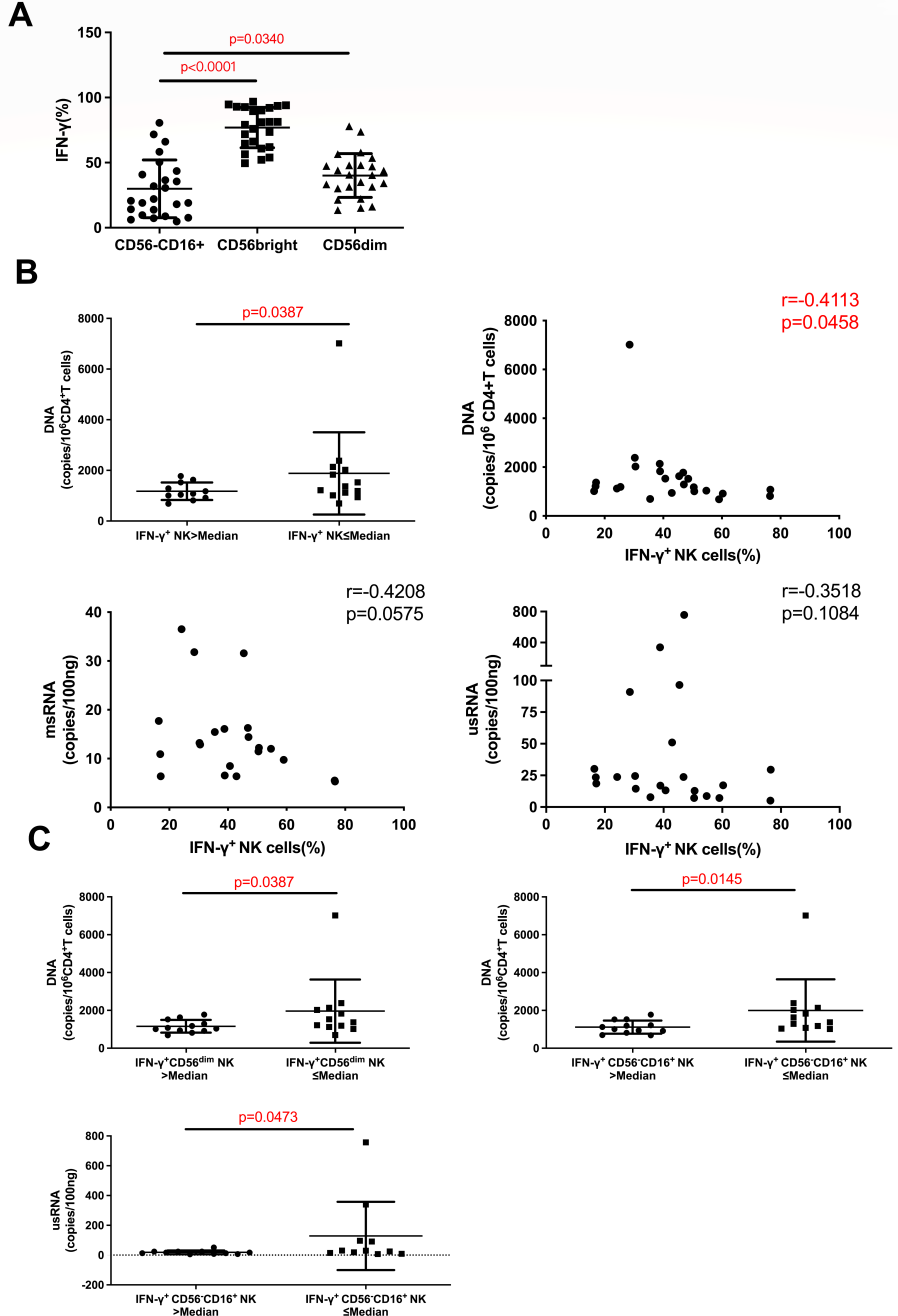
The relationship between the percentage of IFN-γ^+^ NK cells and the reservoir in HIV-infected individuals. (A) Comparison of the level of IFN-γ produced by CD56^−^CD16^+^, CD56^dim^, and CD56^bright^ NK subsets (*n* = 24). (B) Comparison of HIV DNA between HIV-infected individuals with high and low percentages of IFN-γ^+^ NK cells (*n* = 24), relationship analysis between the percentage of IFN-γ^+^ NK cells and HIV DNA (*n* = 24), msRNA (*n* = 21), and usRNA (*n* = 22). (C) Comparison of HIV DNA (*n* = 24) and HIV usRNA (*n* = 22) between HIV-infected individuals with high and low percentages of IFN-γ^+^ CD56^dim^ or IFN-γ^+^ CD56^−^CD16^+^ NK cells. Spearman correlation was used for correlation analysis, the Mann–Whitney *U* test was used for comparison between two groups, Wilcoxon paired rank-sum test was used for comparison between two paired groups.

Upon stimulation, NK cells exhibit potent antiviral and immunoregulatory capabilities through the production of substantial amounts of IFN-γ. Our comparative analysis of IFN-γ production among NK cell subsets and found that the percentage of IFN-γ^+^ CD56^−^CD16^+^ NK cells was significantly lower compared to the percentage of IFN-γ^+^ CD56^bright^ (*P* < 0.0001) and IFN-γ^+^ CD56^dim^ (*P* = 0.0340) NK cells (Fig. 2A). We further examined the effect of IFN-γ production by NK cells on the HIV reservoir and found that HIV-infected individuals with higher percentage of IFN-γ^+^ NK cells had lower HIV DNA copy numbers (*P* = 0.0387); and the percentage of IFN-γ^+^ NK cells was negatively related to HIV DNA copy numbers (*r* = −0.4113, *P* = 0.0458). Although the relationship between IFN-γ and HIV msRNA concentrations did not reach statistical significance, there was a trend toward an inverse association (*r* = −0.4208, *P* = 0.0575) (Fig. 2B). Moreover, our analysis revealed that HIV-infected individuals with high percentage of IFN-γ^+^ CD56^dim^NK cells had a lower number of HIV DNA copies (*P* = 0.0387), while those with high percentage of IFN-γ^+^ CD56^−^CD16^+^ NK cells exhibited lower HIV DNA and usRNA concentrations (*P* = 0.0145, *P* = 0.0473) (Fig. 2C). The correction for multiple testing in correlation analyses with FDR analysis showed that the adjusted *P* value for the correlation between the percentage of IFN-γ^+^NK cells with HIV DNA copy numbers was not significant.

### The expression level of TIGIT on NK cells of HIV-infected individuals has a positive correlation with HIV reservoir

**Fig 3:**
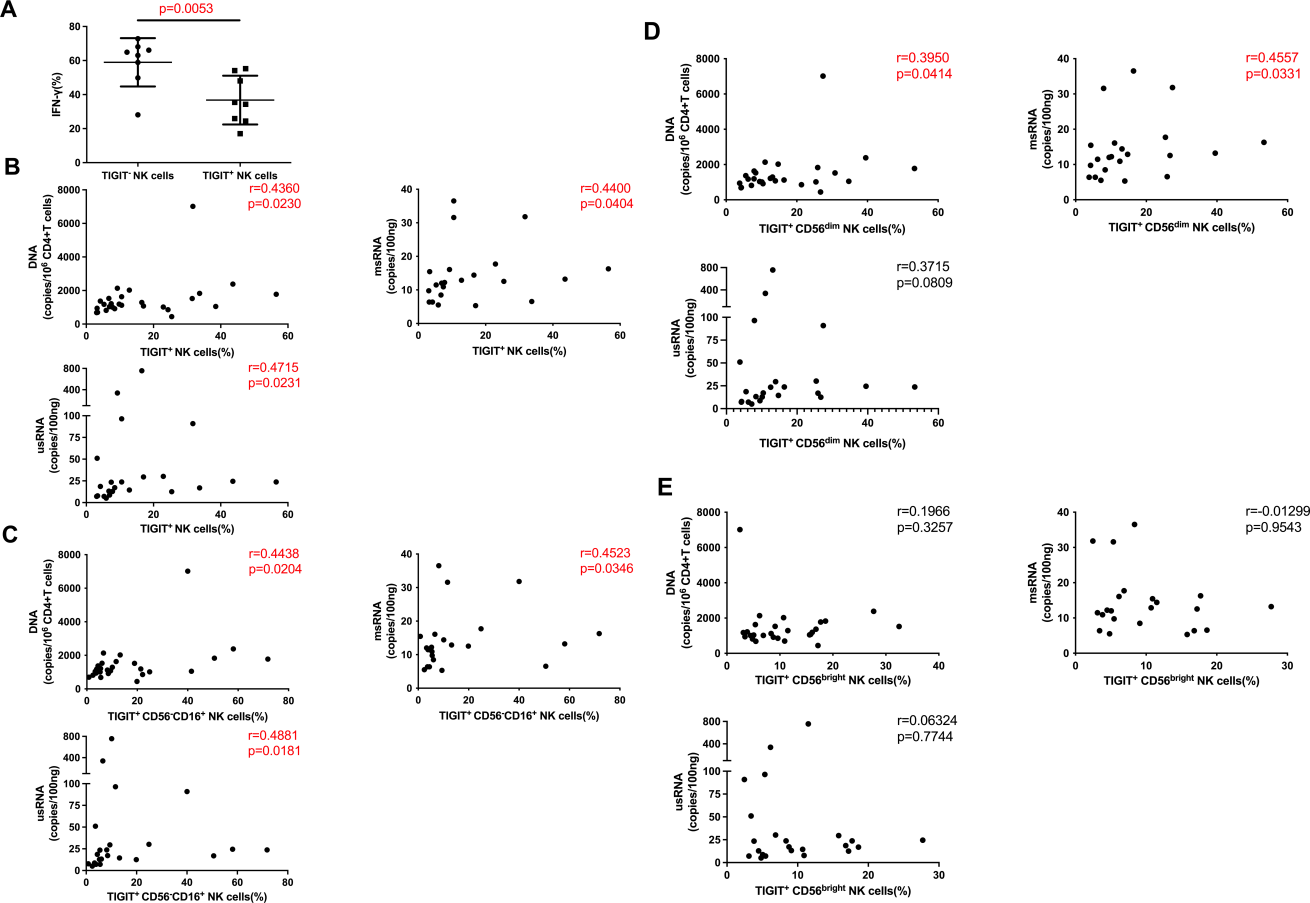
The relationship between TIGIT expression level on NK cells and the reservoir. (A) Comparison of the level of IFN-γ produced by TIGIT^+^ NK cells and TIGIT^−^ NK cells (*n* = 8). (B) Relationship analysis between TIGIT expression level on NK cells and HIV DNA (*n* = 27), msRNA (*n* = 22), and usRNA (*n* = 23). (C) Relationship analysis between TIGIT expression level on CD56^−^CD16^+^ NK cells and HIV DNA (*n* = 27), msRNA (*n* = 22), and usRNA (*n* = 23). (D) Relationship analysis of TIGIT expression level on CD56^dim^ NK cells with HIV DNA (*n* = 27), msRNA (*n* = 22), and usRNA (*n* = 23). (E) Relationship analysis of TIGIT expression level on CD56^bright^ NK cells with HIV DNA (*n* = 27), msRNA (*n* = 22), and usRNA (*n* = 23). Spearman correlation was used for correlation analysis, Wilcoxon paired rank-sum test was used for comparison between two paired groups.

As the most highly expressed immune inhibitory receptor on NK cells, TIGIT exerts a potent regulatory effect on NK cell function. We found that the production of IFN-γ by TIGIT^−^ NK cells was significantly higher than TIGIT^+^ NK cells (*P* = 0.0053) (Fig. 3A). We then analyzed the effect of TIGIT expression on NK cells on the HIV reservoir and indicated that the percentage of TIGIT^+^ NK cells was positively correlated with concentrations of HIV DNA, HIV msRNA, and HIV usRNA (*r* = 0.4360, *P* = 0.0230; *r* = 0.4400, *P* = 0.0404; and *r* = 0.4715, *P* = 0.0231, respectively) (Fig. 3B). Furthermore, we found that the expression level of TIGIT on CD56^−^CD16^+^ NK cells was also positively correlated with concentrations of HIV DNA, HIV msRNA, and HIV usRNA (*r* = 0.4438, *P* = 0.0204; *r* = 0.4523, *P* = 0.0346; and *r* = 0.4881, *P* = 0.0181, respectively) (Fig. 3C). Similarly, the TIGIT expression level on CD56^dim^ NK cells was positively correlated with HIV DNA and HIV msRNA concentrations (*r* = 0.3950, *P* = 0.0414 and *r* = 0.4557, *P* = 0.0331, respectively), and displayed a trend toward positive correlation with HIV usRNA concentrations (*r* = 0.3715, *P* = 0.0809) (Fig. 3D). In contrast, no significant relationship was observed between the expression level of TIGIT on CD56^bright^ NK cells and HIV DNA, HIV msRNA, or HIV usRNA concentrations (Fig. 3E). The correction was done for multiple testing in correlation analyses with FDR analysis, the adjusted *P* values are still significant in Fig.3B through D. Additionally, we found that there was no difference in TIGIT expression between NK cells with high CD57 expression and with low CD57 expression levels (Fig. S1).

### HIV-infected individuals with a lower percentage of TIGIT^+^ NK cells and a higher percentage of CD226^+^ cells exhibit a smaller HIV reservoir

**Fig 4:**
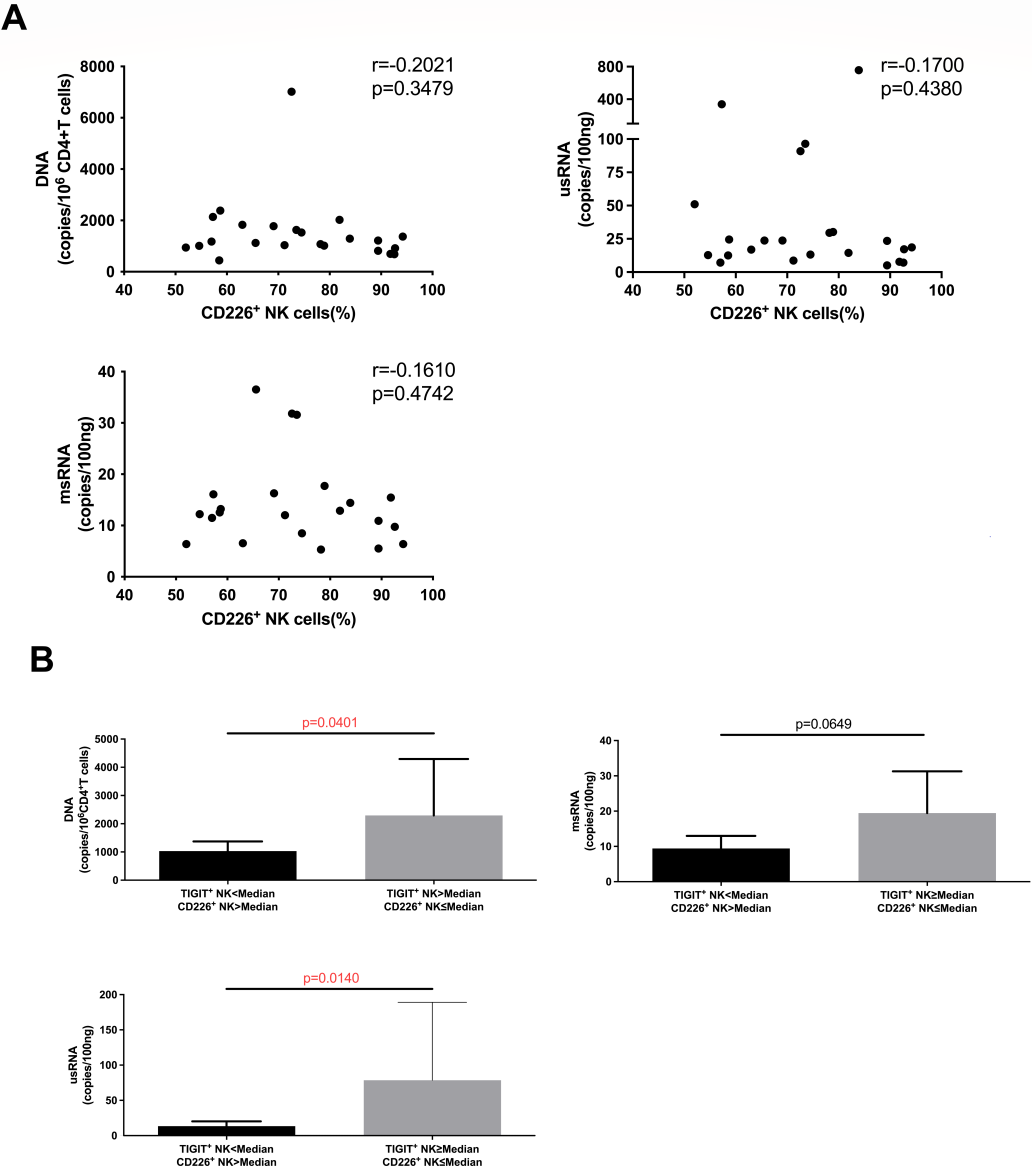
The relationship between the expression of activated receptors on NK cells of HIV-infected individuals and the reservoir. (A) Relationship analysis of CD226 expression level on NK cells with HIV DNA (*n* = 23), msRNA (*n* = 22), and usRNA (*n* = 23). (B) Comparison of HIV DNA (*n* = 15), msRNA (*n* = 12), and usRNA (*n* = 15) between the group with fewer TIGIT^+^ NK cells but more CD226^+^ NK cells and the group with more TIGIT^+^ NK cells but few CD226^+^ NK cells. Spearman correlation was used for correlation analysis, Mann–Whitney *U* test was used for comparison between the two groups.

The killing function of NK cells is regulated by activating receptors such as CD226 and inhibitory receptors such as TIGIT. CD226 and TIGIT compete with each other to bind their shared ligand, CD155. We observed that the expression level of CD226 on NK cells did not significantly correlate with HIV DNA, HIV msRNA, or HIV usRNA concentrations (Fig. 4A). We further analyzed the relationship between the expression levels of TIGIT and CD226 on NK cells and the HIV reservoir, and observed that HIV-infected individuals with a lower percentage of TIGIT^+^ NK cells and a higher percentage of CD226^+^ NK cells exhibited significantly lower concentrations of HIV DNA and HIV usRNA (*P* = 0.0401 and *P* = 0.0140, respectively). Moreover, there was a notable trend, the HIV-infected individuals with a lower percentage of TIGIT^+^ NK cells and a higher percentage of CD226^+^ NK cells had lower concentrations of HIV msRNA (*P* = 0.0649) (Fig. 4B).

### TIGIT can inhibit the PI3K-Akt-mTOR- mTORC1 (s6k) signaling pathway to decrease the production of IFN-γ on NK cells

**Fig 5:**
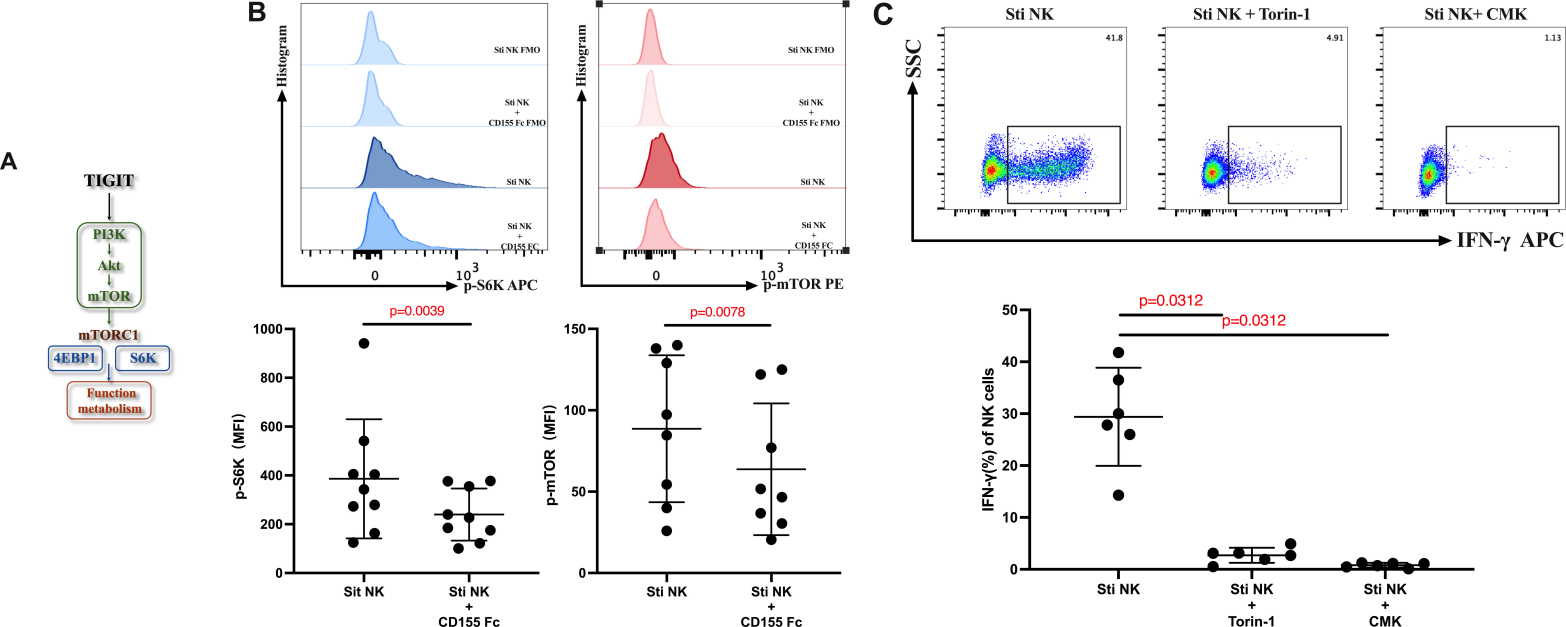
The effect of TIGIT/CD155 on the PI3K-Akt-mTOR pathway of NK cells. (A) Schematic diagram of PI3K-Akt-mTOR pathways. (B) Typical flow diagram and statistical diagram of the effect of TIGIT/CD155 on the PI3K-Akt-mTOR pathway of NK cells (S6k: *n* = 9; mTOR: *n* = 8). (C) Typical flow diagram and Statistical diagram of the effect of Torin1 and CMK on IFN-γ production of NK cells treated with anti-TIGIT (*n* = 6). Wilcoxon paired rank-sum test was used for comparison between the two paired groups.

A previous study in gastric cancer patients demonstrated that TIGIT/CD155 signaling downregulates the PI3K-Akt-mTOR pathway in CD8^+^ T cells, leading to CD8^+^ T cell exhaustion (27). Prompted by this observation, we investigated whether TIGIT similarly modulates NK cell function via the PI3K-Akt-mTOR pathway. We conducted an analysis of the phosphorylation levels of key target proteins within the PI3K-Akt-mTOR signaling cascade downstream of TIGIT activation. mTORC1 includes two target proteins, 4EBP1 and s6k, which play a crucial role in regulating the function and metabolism of NK cells (28) (Fig. 5A). We activated the TIGIT signal by adding CD155-Fc and subsequently observed the phosphorylation levels of mTOR and s6k in NK cells. Our results indicated significant reductions in the phosphorylation of both s6k and mTOR (s6k: *P* = 0.0039; mTOR: *P* = 0.0078) (Fig. 5B). These results revealed that when TIGIT on the surface of NK cells binds to its ligand CD155, it primarily inhibits the PI3K-Akt-mTOR-mTORC1 (s6k) signaling pathway. To further evaluate the effect of the PI3K-Akt-mTOR signaling pathway on NK cell function. We treated NK cells with the mTOR inhibitor Torin1 and the s6k inhibitor CMK, and measured the production of IFN-γ. The results showed that both Torin1 and CMK could significantly inhibit IFN-γ production in NK cells (Torin1: *P* = 0.0312; CMK; *P* = 0.0312) (Fig. 5C). It is shown that TIGIT-mediated inhibition of the PI3K-Akt-mTOR-mTORC1 pathway decreases IFN-γ production in NK cells.

### TIGIT inhibition on NK cells enhances the ability of NK cells to suppress HIV reservoir

**Fig 6:**
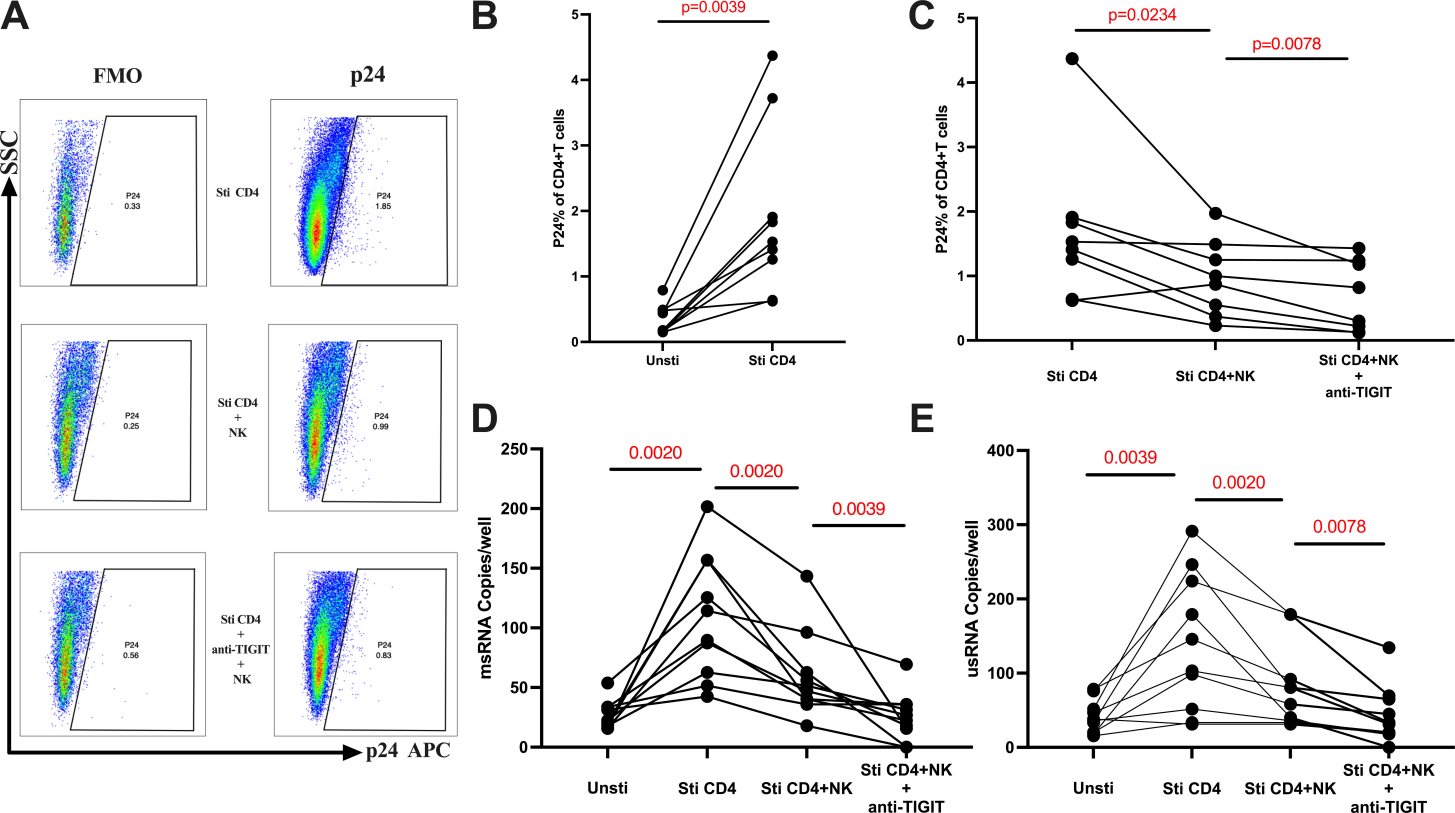
The comparison of P24 expression levels, HIV msRNA, and HIV usRNA of CD4^+^ T cells in latent HIV-infected CD4^+^ T cells suppression assay. (A) Typical flow diagram for flow cytometry of detection of P24 expression levels of CD4^+^ T cells. (B) Comparison of P24 expression levels of CD4^+^ T cells between unstimulated and stimulated CD4^+^ T cells (*n* = 9). (C) Comparison of P24 expression levels of CD4^+^ T cells among three groups: stimulate, incubate with NK cells, and incubate with NK cells pretreat with anti-TIGIT antibody (*n* = 8). (D) Comparison of HIV msRNA concentrations among four groups: un-stimulate, stimulate, incubate with NK cells, and incubate with NK cells pretreat with anti-TIGIT antibody (*n* = 10). (E) Comparison of HIV usRNA concentrations among four groups: un-stimulate, stimulate, incubate with NK cells, and incubate with NK cells pretreat with anti-TIGIT antibody (*n* = 10). Wilcoxon paired rank-sum test was used for comparison between the two paired groups.

To explore the ability of NK cells to suppress HIV reservoir after blocking TIGIT on NK cells, we examined the levels of intracellular p24 in latent HIV-infected CD4^+^ T cells suppression assay. We illustrated the gating strategy and typical flow diagram employed to detect the p24 levels of CD4^+^ T cells (Fig. S2; Fig. 6A). The results showed that CD4^+^ T cells from the individuals who have undergone ART for over 2 years were activated, and the expression levels of intracellular p24 were significantly increased (*P* = 0.0039) (Fig. 6B). While significantly reduced upon the addition of autologous NK cells (*P* = 0.0234). Furthermore, compared to the addition of autologous NK cells alone, the intracellular p24 levels in CD4^+^ T cells are even more significantly reduced when the autologous NK cells are pretreated with anti-TIGIT blocking antibodies (*P* = 0.0078) (Fig. 6C).

In addition to assessing the intracellular p24 levels in CD4^+^ T cells, we also compared the concentrations of HIV msRNA and usRNA among different groups. After co-incubation of CD4^+^ T cells with autologous NK cells or NK cells pretreated with anti-TIGIT blocking antibody. We found that the HIV msRNA significantly increased upon CD4^+^ T cell activation (*P* = 0.002), but significantly reduced following the addition of autologous NK cells (*P* = 0.002). Compared to the addition of autologous NK cells alone, the HIV msRNA is further significantly reduced when the autologous NK cells are pretreated with anti-TIGIT blocking antibodies (*P* = 0.0039) (Fig. 6D). Subsequent measurements of HIV usRNA yielded similar results, with a significant increase upon CD4^+^ T cell activation (*P* = 0.0039), followed by a significant reduction upon the addition of autologous NK cells (*P* = 0.002). Furthermore, the HIV usRNA is even more significantly reduced when adding autologous NK cells pretreated with anti-TIGIT blocking antibodies compared to the addition of untreated autologous NK cells (*P* = 0.0078) (Fig. 6E).

## DISCUSSION

NK cells do not necessitate stimulation by specific antigens and their function is regulated through the coordinated action of surface-expressed activating and inhibitory receptors (29). To gain a deeper understanding of the key factors influencing the HIV reservoir and explore potential eradication strategies, it is crucial to conduct in-depth research into the relationship between NK cells and the HIV reservoir. In this study, the reservoir indicators were HIV DNA, and HIV usRNA and msRNA copy numbers. The inclusion of usRNA and msRNA concentrations in the analysis was crucial due to the presence of defective HIV genes within the viral DNA, which cannot undergo transcription and translation, potentially leading to an overestimation of the viral reservoir size. HIV usRNA levels serve as a valuable metric for assessing the *in vivo* activation of the latent HIV reservoir. This biomarker has recently gained prominence in clinical trials, where it is being utilized to monitor the progress of HIV reservoir elimination strategies (30). The abundance of HIV msRNA within infected cells is indicative of their potential to engage in viral replication (31). Defective HIV genomes often harbor deletions in the TAT and REV genes, making it challenging for cells harboring such viruses to produce corresponding transcripts (32).

Our study found that the group of HIV-infected individuals with higher percentage of CD56^dim^ and CD56^bright^ NK cells had lower HIV DNA copy numbers. CD56^dim^ NK cells are known for their potent cytotoxic capabilities, utilizing granzyme B and perforin to eliminate infected cells (33). In contrast, CD56^bright^ NK cells exhibit a lower cytotoxic potential but possess the ability to secrete antiviral and immunomodulatory cytokines, thereby modulating the immune response(34); both of these NK cell subsets play an important antiviral role. Our study further revealed a positive correlation between the percentage of CD56^−^CD16^+^ NK cells and HIV DNA copy numbers in HIV-infected individuals. A previous study reported that the percentage of CD56^−^CD16^+^ NK cells gradually increased during the chronic stages of HIV infection (35). This NK cell subset is characterized by its high production of IL-10 and tumor growth factor-β, cytokines that play a pivotal role in suppressing the function of CD8^+^ T cells, thus classifying it as a negative regulatory subset (18). Notably, our investigation also indicated that among the three NK cell subsets examined, CD56^−^CD16^+^ NK cells produced the lowest levels of IFN-γ.

Our study showed that the percentage of IFN-γ^+^ NK cells was negatively correlated with HIV DNA copy numbers, suggesting a similar negative trend with respect to HIV msRNA concentrations. In addition, the group that possessed a higher percentage of IFN-γ^+^ NK or IFN-γ^+^CD56^dim^ NK cells exhibited significantly lower copy numbers of HIV DNA. Similarly, those with a higher percentage of IFN-γ^+^CD56^−^CD16^+^ NK cells exhibited lower concentrations of HIV DNA and HIV usRNA. One study found that the ability of NK cells to clear the virus was significantly compromised in mice after knocking out the *Ifn* gene, and the IFN-γ was demonstrated to exert a direct antiviral effect in mice infected with lymphocytic choriomeningitis virus (36). Thus, IFN-γ is an important anti-virus cytokine that can clear infected cells in a non-toxic manner. IFN-γ exerts antiviral effects by binding to cell surface receptors, which in turn triggers the phosphorylation of Jak1 and Jak2, thereby activating the JAK/STAT pathway (37). Subsequently, the activated protein kinases recruit and phosphorylate cytoplasmic STAT proteins. Those proteins then transform into transcription factor complexes (38, 39), which stimulate IFN-stimulated genes (ISGs) such as those encoding the IRF7, 2′,5′-oligoadenylate synthetase 1 (OAS1), protein kinase R (PKR), and myxovirus resistance protein A (MxA) (40). Thus, NK cells not only reduce HIV DNA copy numbers but also limit the size of the active HIV reservoir. Our study employed a cytokine stimulation protocol to evaluate NK cell function has potential limitations. One study found that combined stimulation with IL-12/15/18 does not activate LFA-1 (41), which is crucial for stable contact between NK cells and target cells (42) and the polarization of lytic granules toward NK cell target (43), suggesting that cytokine stimulation protocol is not suitable for assessing NK cell cytotoxicity. Nevertheless, numerous previous studies have demonstrated that IL-12/15/18 plays indispensable roles in the production of cytokines by NK cells (44–47). In our study, IL-12/15/18 can be used to assess the production of IFN-γ by NK cells.

This study showed that TIGIT^+^ NK cells produce less IFN-γ than TIGIT^−^ NK cells, while one study found that TIGIT^+^ NK cells produce more IFN-γ than TIGIT^−^ NK (48). However, numerous other studies consistently found that TIGIT inhibits the expression of TNF-α, CD107a, and IFN-γ in NK cells, which aligns with the observation in our study that TIGIT^+^ NK cells exhibit reduced IFN-γ production (49–51). Our analysis noted that the expression of TIGIT on NK cells and the CD56^−^CD16^+^ NK cell subset was significantly positively correlated with HIV DNA, HIV msRNA, and HIV usRNA concentrations. In addition, the expression of TIGIT on CD56^dim^NK cells was significantly positively correlated with HIV DNA and HIV msRNA concentrations and showed a tendency toward positive correlation with HIV usRNA concentrations. Our previous report that TIGIT expression was upregulated on the CD56^−^CD16^+^ and CD56^dim^ NK cell subsets following HIV infection, whereas no significant change was noted in CD56^bright^ NK cells, and also found that the CD155 which is TIGIT ligand on CD4^+^ T cells was upregulated after HIV infection (23). Notably, even under ART, some latently infected cells remain incompletely silenced, maintaining active gene expression (52). These transcriptionally active HIV-infected cells are detectable by the immune system, including NK cells. Nevertheless, the elevated expression of TIGIT on NK cells potentially impedes their function through the TIGIT/CD155 interaction, thereby compromising NK-mediated efforts to eliminate the HIV reservoir. Interestingly, while CD226 expression on NK cells alone did not correlate with the HIV reservoir in our study, a combined analysis of TIGIT and CD226 revealed a pattern: HIV-infected individuals with a lower percentage of TIGIT^+^ NK cells and a higher percentage of CD226^+^ NK cells exhibited lower concentrations of HIV DNA and HIV usRNA. Given that CD155 also serves as a ligand for CD226 (53), and considering the high expression of TIGIT on CD226^+^ NK cells (23), it is plausible that TIGIT may competitively inhibit the interaction between CD226 and CD155. In fact, the TIGIT/CD155 interaction is stronger than the CD226/CD155 interaction (54). These findings imply that excessive TIGIT expression may hinder the ability of CD226 to function as an activating receptor on NK cells, ultimately weakening the NK cell-mediated immune response against HIV infection. It should be noted that the HIV DNA copies of one patient in our results are too high, which may skew the relevant results. Nevertheless, this patient’s test result is authentic and accurate, and we cannot arbitrarily exclude it from our research cohort. Therefore, when faced with this part of the results, we should approach them with caution.

Our research revealed that activation of TIGIT could reduce the phosphorylation levels of mTOR and s6k in NK cells, and inhibition of mTOR and s6k phosphorylation could further impede the production of IFN-γ by NK cells. These findings indicated that TIGIT regulates the function of NK cells through the PI3K-Akt-mTOR-mTORC1 (s6k) pathway, and may potentially affect the control of NK cells in the HIV reservoir. TIGIT can recruit Grb2 through ITTF-like motifs, which subsequently facilitate the recruitment of SHIPI (55), and inhibitor of the PI3K signaling pathway (56). The PI3K-AKT-mTOR Pathway is essential for NK cell development and activation (57). In previous reports, mice infected with MCMV and treated with the mTOR inhibitor rapamycin exhibited impaired proliferation and reduced IFN-γ production by NK cells (58, 59). Similar effects were observed when PI3K and AKT inhibitors were utilized (60). This recruitment of SHIPI by TIGIT serves to inhibit the PI3K-AKT-mTOR pathway, ultimately leading to a further suppression of IFN-γ production by NK cells. These findings underscore the intricate regulatory mechanisms involving TIGIT and its downstream effects on NK cell function, which may have important implications for immune responses to viral infections, including HIV.

To further investigate the potential role of TIGIT in HIV reservoir control, we performed *in vitro* experiments that demonstrated that blocking TIGIT significantly enhanced the ability of NK cells to suppress the HIV reservoir. These results, in conjunction with our previous findings, underscored TIGIT as an important factor affecting NK cell cytotoxicity. Therefore, reducing TIGIT expression on NK cells would be essential for HIV reservoir eradication.

In summary, our findings underscore the critical role of NK cells in controlling the HIV reservoir. We observed that the percentage of NK subsets, their IFN-γ production, and TIGIT expression characteristics were intimately linked to the HIV reservoir. Importantly, TIGIT was found to inhibit the PI3K-Akt-mTOR-mTORC1 signaling pathway, leading to decreased IFN-γ production by NK cells. Blocking TIGIT significantly enhanced NK cell function, resulting in reduced numbers of reactivated latently infected cells and lower concentrations of HIV msRNA and usRNA. Based on these findings, we speculate that NK cells may serve as potent effector cells in HIV reservoir clearance. Targeted therapies aimed at inhibiting TIGIT expression on NK cells could potentially shrink the HIV reservoir and pave the way for novel strategies in HIV cure research.

## SUPPLEMENTAL MATERIAL

Fig. S1 mbio.03226-24-s0001.tiff The relationship between the expression of TIGIT and CD57 on NK cells.

Fig. S2 mbio.03226-24-s0002.tiff The gating strategy of flow cytometry for detecting p24 expression in CD4+ T cells.

Supplemental material mbio.03226-24-s0003.docx Tables S1 to S3; legends for Fig. S1 and S2.

ASM does not own the copyrights to Supplemental Material that may be linked to, or accessed through, an article. The authors have granted ASM a non-exclusive, world-wide license to publish the Supplemental Material files. Please contact the corresponding author directly for reuse.
