# Supplementary figures and images for: Inhibition of TIGIT on NK cells improves their cytotoxicity and HIV reservoir eradication potential
Source: mBio. 2025 Feb 7;16(3):e03226-24. doi: 10.1128/mbio.03226-24 (PMC11898710; doi:10.1128/mbio.03226-24)

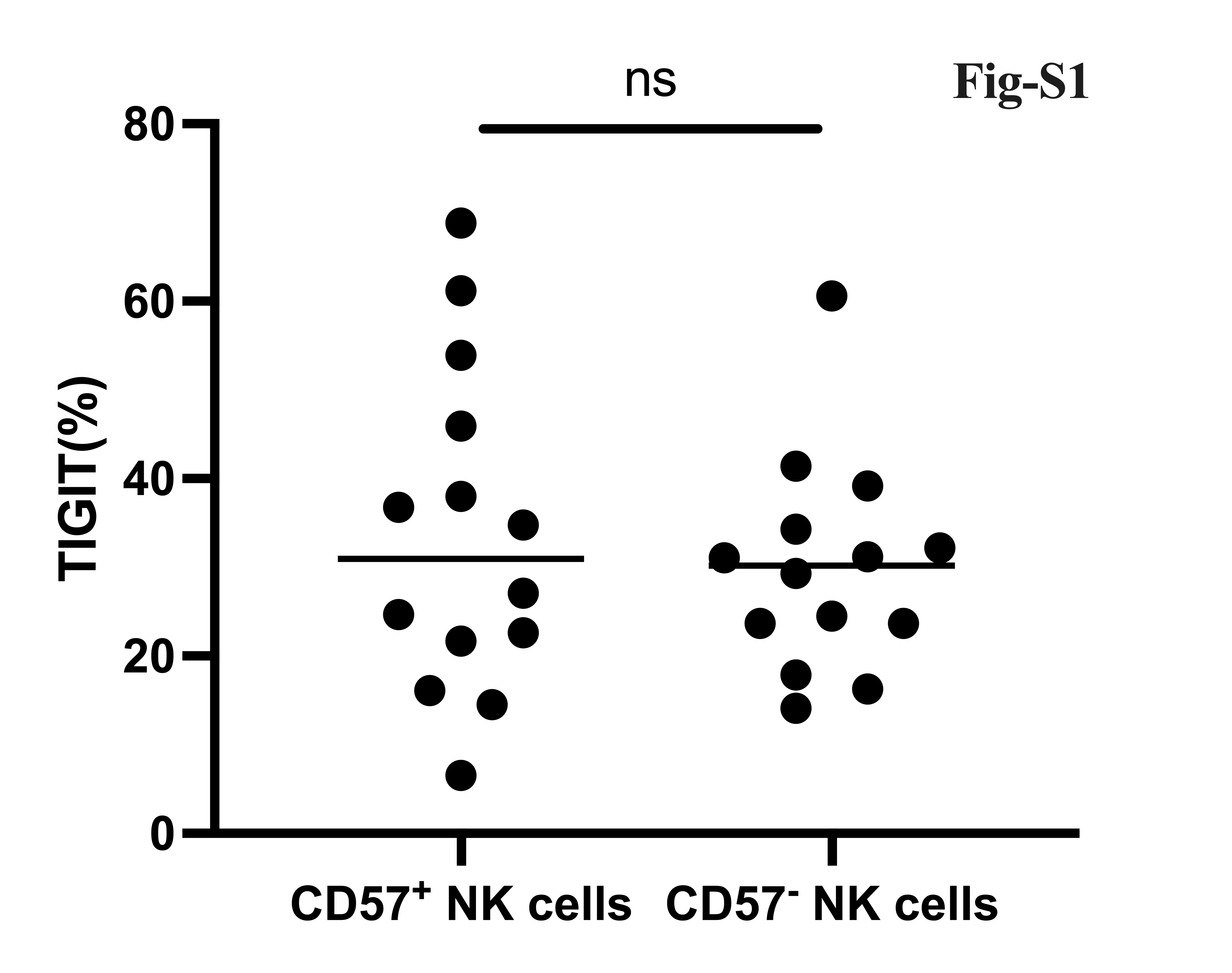

Supplement: Fig. S1 — The relationship between the expression of TIGIT and CD57 on NK cells. [file mbio.03226-24-s0001.tiff]

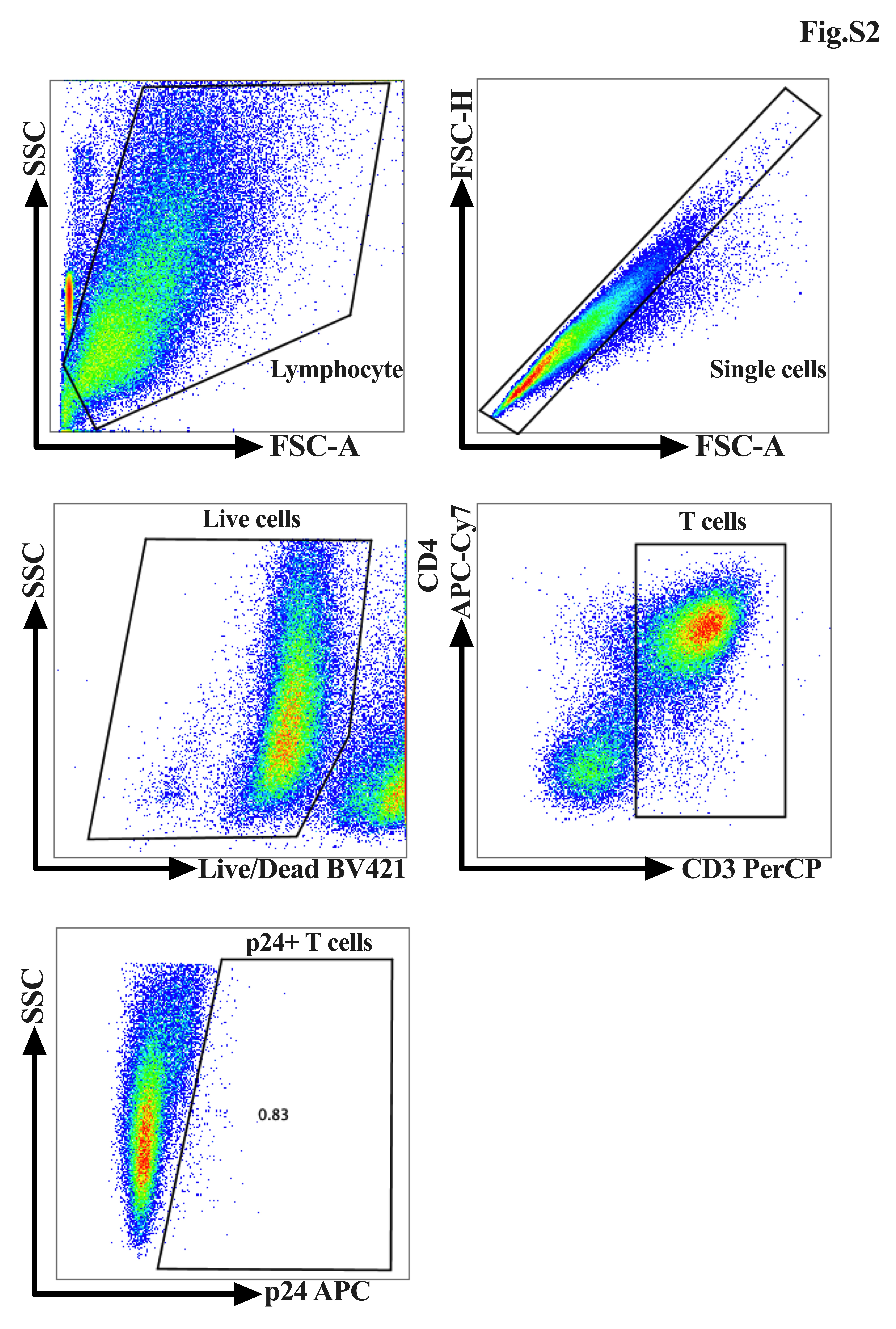

Supplement: Fig. S2 — The gating strategy of flow cytometry for detecting p24 expression in CD4+ T cells. [file mbio.03226-24-s0002.tiff]
