# Supplementary material for: Inhibition of TIGIT on NK cells improves their cytotoxicity and HIV reservoir eradication potential
Source: mBio. 2025 Feb 7;16(3):e03226-24. doi: 10.1128/mbio.03226-24 (PMC11898710; doi:10.1128/mbio.03226-24)
Supplement: Supplemental material — Tables S1 to S3; legends for Fig. S1 and S2. [file mbio.03226-24-s0003.docx]

**Supplementary Materials**

| Reagents | Volume（1×） |
| --- | --- |
| 20×iScript Advanced Reverse Transcriptase | 1μl |
| 5×iScript Advanced Reaction Mix | 4μl |
| RNA sample | 15μl |
| Total volume | 20μl |

**Table S1.** Reverse transcription reaction system

**Table S2.** Digital droplet PCR reaction system

| Reagents | RPP30(μl) | LTRG(μl) | Tat-Rev(μl) | Gag(μl) |
| --- | --- | --- | --- | --- |
| ddPCR supermix for probes | 10 | 10 | 10 | 10 |
| Probe(5uM/L) | 1 | 1 | 1 | 1 |
| Forward Prime(10uM/L) | 0.75 | 0.75 | 0.75 | 0.75 |
| Reverse Prime (10uM/L) | 0.75 | 0.75 | 0.75 | 0.75 |
| Nuclease-Free Water | 7.5 | 4.5 | 4.5 | 4.5 |
| Sample | 1 | 4 | 4 | 4 |
| Total volume | 21 | 21 | 21 | 21 |

**Table S3.** Digital droplet PCR primer and probe list

| Probe | RPP30 | FAM- CTG ACC TGA AGG CTC T-TAMRA |
| --- | --- | --- |
|  | LTRG | FAM- CTC TCT CCT TCT AGC CTC-TAMRA |
|  | Tat-Rev | FAM-AGGGGACCCGACAGGCCC-TAMRA |
|  | Gag | FAM-GACCATCAATGAGGAAGCTGCAGAATGGGAT  -TAMRA |
| Prime | RPP30-F | GATTTGGACCTGCGAGCG |
|  | RPP30-R | GCGGCTGTCTCCACAAGT |
|  | LTRG-F | TACTGACGCTCTCGCACC |
|  | LTRG-R | TCTCGACGCAGGACTCG |
|  | Tat-Rev-F | CTTAGGCATCTCCTATGGCAGGA |
|  | Tat-Rev-R | GGATCTGTCTCTGTCTCTCTCTCCACC |
|  | Gag-F | AGTTGGAGGACATCAAGCAGCCATGCAAAT |
|  | Gag -R | TGCTATGTCAGTTCCCCTTGGTTCTCT |

**FIG S1.** **The relationship between the expression of TIGIT and CD57 on NK cells**.

S1: Comparison of the expression level of TIGIT between CD57^+^ NK cells and CD57^-^ NK cells(n=14).

**FIG S2.** **The gating strategy of flow cytometry for detecting p24 expression in CD4^+^ T cells.**
